# Identification and verification of biomarkers associated with neutrophils in acute myocardial infarction: integrated analysis of bulk RNA-seq, expression quantitative trait loci, and mendelian randomization

**DOI:** 10.3389/fmolb.2025.1614350

**Published:** 2025-08-08

**Authors:** Guoqing Liu, Xiangwen Lv, Jiahui Qin, Xingqing Long, Miaomiao Zhu, Chuwen Fu, Jian Xie, Peichun He

**Affiliations:** ^1^ Department of Cardiology, The First Affiliated Hospital of Guangxi Medical University, Nanning, Guangxi, China; ^2^ Department of Cardiology, The Second Affiliated Hospital of Guangxi Medical University, Nanning, Guangxi, China; ^3^ The First Affiliated Hospital of Guangxi Medical University, Nanning, Guangxi, China; ^4^ School of Basic Medical Sciences, Guangxi Medical University, Nanning, Guangxi, China

**Keywords:** acute myocardial infarction, neutrophils, mendelian randomization, immune infiltration, expression quantitative trait loci

## Abstract

**Background:**

Immune infiltration is closely related to the progression of acute myocardial infarction (AMI), among which neutrophils have received extensive attention. However, the concrete association between AMI and neutrophils remains uncertain.

**Methods:**

Bulk RNA-seq data for patients with AMI were downloaded from the Gene Expression Omnibus (GEO) database. CIBERSORT was utilized to measure 22 degrees of immune cell composition. The causal link between neutrophils and AMI was determined by Mendelian randomization (MR) analysis. Genes with correlation coefficients >0.7 with neutrophils were selected, and their representativeness was confirmed by functional enrichment analysis. Weighted gene co-expression network analysis (WGCNA) was performed to screen for AMI-related modular genes. Robust molecular clusters linked to neutrophils were recognized via consensus clustering methodology. Hub genes were screened using the least absolute shrinkage and selection operator (LASSO) and random forest (RF) algorithms. A cellular model of AMI was established using oxygen- and glucose-deprived AC16 cells. Quantitative reverse transcription‒polymerase chain reaction (RT‒qPCR) was used to validate the gene expression levels. The expression quantitative trait loci (eQTL) analysis is used to identify genetic variations in the expression of regulatory genes in AMI.

**Results:**

MR results demonstrated a significant causal relationship between neutrophils and AMI. The consensus clustering method delineated two gene subclusters, and the expression of AMI-related neutrophil coexpressed genes was consistent with innate immune cell infiltration. Three hub neutrophil coexpressed genes (*BCL6*, *CDA*, and *IL1R2*) were identified. The receiver operating characteristic (ROC) curves indicated that the three genes were valuable for diagnosing AMI in the training and validation sets, and the RT‒qPCR results verified the gene expression data. A prediction model was constructed based on three hub neutrophil coexpressed genes in AMI, and the results revealed good accuracy. The eQTL analysis further confirmed that *BCL6* plays a pivotal role as a key risk gene in neutrophil-mediated damage in AMI.

**Conclusion:**

There is a causal relationship between neutrophils and AMI. *BCL6* plays a pivotal role as a key risk gene in neutrophil-mediated damage in AMI. However, more comprehensive studies are needed to determine the molecular mechanism of AMI-related neutrophil coexpressed genes.

## Introduction

Acute myocardial infarction (AMI) is a disease with high morbidity and mortality caused by myocardial necrosis due to coronary artery obstruction (Li et al., 2024). Rupture or erosion of an atherosclerotic plaque is the most frequent cause of AMI; other causes include coronary spasm, embolism, and entrapment (Krittanawong et al., 2023). Rapid restoration of myocardial blood flow is a crucial therapeutic factor in the prognosis of AMI, and conventional treatments include thrombolysis and percutaneous coronary intervention (Chen et al., 2023). However, drugs are prone to side effects such as bleeding and gastrointestinal adverse effects, and interventional or surgical interventions not only need to be alert to complications such as arrhythmia and pericardial effusion but also need to prevent ischemia‒reperfusion injury and coronary restenosis (Zhang et al., 2023). All these risk factors threaten patient prognosis. Thus, investigating the mechanisms underlying AMI incidence and developing cutting-edge therapeutic approaches are essential.

The mechanisms of AMI are complex and involve various pathological states and signaling pathways, such as oxidative stress, calcium overload, and ferroptosis. Immune infiltration is an essential part of this process, especially in coordinating tissue remodeling. The mean neutrophil volume was found to be positively correlated with the function of the left ventricle and infarct area by van Hout et al. (2015). After acute infarction, the innate immune response in the infarcted myocardium induces the release of damage associated molecular patterns (DAMPs), which bind to pattern-recognition receptors, including Toll-like receptors (TLRs) and NOD-like receptors, and promote the expression of chemokines. Neutrophils rapidly aggregate into the damaged myocardium, polarize to an inflammatory phenotype and undergo degranulation, the release of reactive oxygen species (ROS), neutrophil extracellular traps (NETs), and the secretion of diverse cytokines (Han et al., 2023). NETs are chromosomal structures released by neutrophil plasma membrane disruption in response to stimulation and are modified by specific cytoplasmic and granular proteins (Sollberger et al., 2018). NETs have prothrombotic molecules that can directly combine with fibrinogen and promote plaque surface erosion while accelerating thrombosis by promoting platelet and erythrocyte aggregation binding and thrombin generation. Neutrophils also seem to have dual roles in further recruiting additional immune cells and stimulating macrophages to polarize toward an anti-inflammatory phenotype and participate in chemokine clearance (Kologrivova et al., 2021). In addition, macrophages and monocytes play essential roles in the inflammatory, proliferative, and maturational phases of AMI, resulting in a proinflammatory phenotype in the early stages of inflammation, followed by a gradual shift to an anti-inflammatory phenotype to promote repair (Jian et al., 2023). Early recruitment of monocytes from the circulation would, in turn, further promote neutrophil aggregation in the infarcted myocardium. Overall, neutrophils initiate the early proinflammatory response in AMI, influencing the prognosis of patients with AMI; thus, therapeutic strategies targeting neutrophils have implications.

**FIGURE 1:**
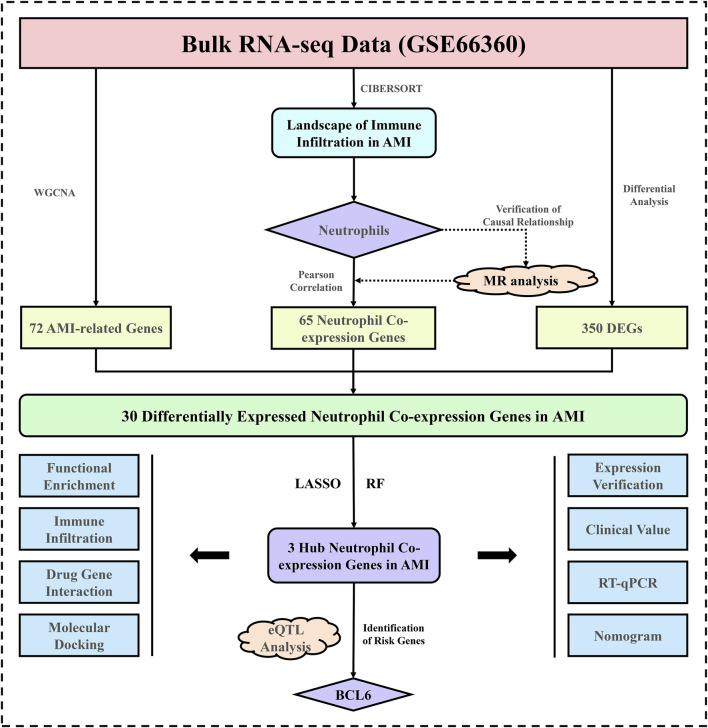
Research flowchart. AMI, acute myocardial infarction; MR, Mendelian randomization; DEGs, differentially expressed genes; LASSO, least absolute shrinkage and selection operator; RF, random forest; RT‒qPCR, quantitative reverse transcription‒polymerase chain reaction; eQTL, expression quantitative trait loci.

Mendelian randomization (MR) is a technique for assessing causal effects due to modifiable nongenetic exposures through genetic data, which is effective in controlling for confounding factors and valuable in inferring disease etiology. This study comprehensively utilized bulk RNA-seq, expression quantitative trait loci (eQTL), and the MR method to identify and validate neutrophil biomarkers associated with AMI. Figure 1 shows the detailed flow of this study, which aimed to explore in depth the mechanism of neutrophil-mediated injury in AMI, laying a theoretical foundation for the development of novel AMI treatment methods.

## Materials and methods

### Data acquisition

The gene expression data (GSE66360 and GSE48060) and platform data (GPL570) were downloaded from the Gene Expression Omnibus (GEO) database, and 71 normal and 80 AMI samples were acquired. GSE66360 contains 50 normal and 49 AMI samples, which function as the training set, whereas GSE48060 serves as the testing set, including the remaining 21 normal and 31 AMI samples.

The eQTL (containing 19,942 genes), neutrophil (ukb-d-30140_irnt [*N* = 349,856]) and myocardial infarction (MI) (ukb-d-I9_MI [*N* = 361,194]) data were collected from the OpenGWAS project (https://gwas.mrcieu.ac.uk/). The above datasets are derived from the European population and have no sex limitations. All summary statistics from the genome-wide association study (GWAS) utilized in this study are publicly accessible and were approved ethically during the initial analysis phase.

### Immune infiltration analysis

We applied CIBERSORT to calculate the composition and content of 22 immune cells in the individual samples. The Pearson correlation test was used to examine the relationships between 22 immune cell types. The bar plot shows how many different immune cells were present in each AMI sample.

### MR analysis

In this study, eQTL and neutrophil GWAS data were used as the exposure data, and MI GWAS data were used as the outcome. The single nucleotide polymorphisms (SNPs) with strong associations (*P* < 5.0 × 10^−8^) were used as instrumental variables (IVs) (Purcell et al., 2007). The linkage disequilibrium settings were adjusted at *R* ^*2*^ < 0.001 and clumping distance = 10,000 kb (Kuppa et al., 2023). SNPs with weak relationships or insufficient explanation of phenotypic variance were eliminated using the filter “F test value > 10”(Bowden et al., 2016). We regarded genetic variance as an IV for MR analysis to estimate the causality between exposure and outcome. Inverse variance weighting (IVW) was the primary MR analysis approach. We adopted additional algorithms to test the robustness of the results, including MR‒Egger, weighted median, maximum likelihood method, and MR-PRESSO. MR‒Egger analysis can be based on the intercept of the IVs to assess whether IVs have a directional horizontal pleiotropic effect on the outcome, the weighted median method allows for valid causal estimation, the maximum likelihood method enhances the statistical efficacy by optimizing the model fitting process through parameters, and MR-PRESSO can remove outliers to correct for horizontal pleiotropy (Verbanck et al., 2018). Multiple method validation will significantly strengthen the robustness of causal inferences. To assess whether individual SNPs affected outcomes, we also performed omission-sensitivity analyses. In this study, we removed SNPs one by one using leave-one-out analysis. We individually calculated the combined effects of the remaining SNPs to ascertain the specific impact of each on the results. If the findings from the leave-one-out analysis diverge from those of the causal effects analysis, it may suggest potential biases in the estimated causal effects. All MR analyses conducted in this study relied on the “TwoSampleMR”, “MendelianRandomization”, “MR-PRESSO”, and “MR.raps” packages in R.

### Acquisition and functional enrichment analysis of neutrophil coexpressed genes

Pearson correlation analysis revealed genes with correlation coefficients >0.7 associated with neutrophil expression levels. Gene Ontology (GO) enrichment analysis was performed to analyze the roles of neutrophil coexpressed genes. Moreover, the Kyoto Encyclopedia of Genes and Genomes (KEGG) was used to explore the roles of genes in multiple fields. We carried out GO and KEGG analyses using the “clusterProfiler”, “org.Hs.eg.db”, “GOplot”, and “enrichplot” packages in R to verify whether we screened the genes for representativeness.

### Identification of gene modules associated with AMI

Weighted gene co-expression network analysis (WGCNA) was used to determine the linkages between gene patterns and sample phenotypes. The top 5,000 genes with average expression values among the 99 samples (GSE66360 dataset) were used for WGCNA to reduce the computational effort. The “WGCNA” package was used to load the gene data, extract the expression data, delete the unwanted data to regenerate the matrix, check for missing values and identify outliers, load the phenotypic data, visualize the linkages between the phenotypic data and the gene expression data, and reconstruct the sample clustering tree. A soft threshold with potential values ranging from 1 to 30 was established by choosing the soft threshold function. We built a weighted gene coexpression network using the “blockwiseModules” function with a modular number of genes ≥60, a soft threshold was determined, and a hierarchical clustering dendrogram was created based on the nearly scale-free topology criterion. We quantified the similarity of all genes on the matrix using quantitative measurements of module membership (MM), which was defined as the correlation between module eigengenes (MEs) and gene expression profiles. Genetic significance (GS) and MM measurements were employed to locate genes strongly linked to AMI, and scatter plots of gene significance related to module membership were drawn.

### Identification of differentially neutrophil coexpressed genes in AMI

We applied thresholds of |log2-fold change (FC)| ≥ 1 and *P* < 0.05. We analyzed the differences in gene expression between the control and AMI groups using the “limma” package. A Venn diagram of the intersection of neutrophil coexpressed genes, module genes, and differentially expressed genes (DEGs) was subsequently drawn, and differentially expressed neutrophil coexpressed genes were screened.

### Molecular typing and immune infiltration

The “ConsensusClusterPlus” package was used for the consensus clustering analysis. We chose the optimal number of clusters by comparing the changes in the area under the cumulative distribution function (CDF) curve for different values of k. The k value representing the number of clusters ranged from 2 to 9. The “ggplot2” and “pheatmap” packages in R were applied to visualize the expression levels of candidate genes between different gene clusters. Afterward, the “prcomp” function of the “stats” package was utilized to confirm the appropriateness of the classification by principal component analysis (PCA). We also performed single-sample genome enrichment analysis (ssGSEA) on the AMI genotyping results and then ranked and summarized the gene expression levels in the samples. After consolidating the results, we determined the immune cell abundance in each sample and investigated the relationships between these AMI-related neutrophil coexpressed genes and immune cells.

### Identification and verification of hub neutrophil coexpressed genes in AMI

Two algorithms were applied to choose appropriate genes. The least absolute shrinkage and selection operator (LASSO) is a data mining tool that introduces a penalty function in the commonly used multiple linear regression. It compresses the coefficients and streamlines the model, thus avoiding covariance and overfitting. Random forest (RF) employs decision trees to evaluate the significance of variables by assigning a value to each one. A threshold of mean decrease in Gini (MDG) > 2.0 was used to screen for core genes that contributed significantly to RF classification. We took the intersection of the above algorithms to obtain the hub neutrophil coexpressed genes in AMI. To evaluate the prediction accuracy of the hub genes, receiver operating characteristic (ROC) curves for every gene in the training and validation cohorts were created. The area under curve (AUC) value of the ROC curve was calculated, and the AUC was 0–1, with a larger AUC value representing better gene prediction performance.

### Gene set enrichment analysis (GSEA) and gene set variation analysis (GSVA) of hub neutrophil coexpressed genes in AMI

GSEA is a data analysis approach that leverages a predefined set of genes to evaluate trends in their distribution within a ranked list of genes based on their phenotypic relevance, thereby determining their collective contribution to the observed phenotype (Wang et al., 2023). We utilized the “ClusterProfiler” package to carry out GSEA. Hub genes were classified into high- or low-expression groups according to the median expression level of the gene. The enrichment pathways of the KEGG reference gene set were downloaded from the MSigDB database. In addition, GSVA is a nonparametric, unsupervised technique for assessing transcriptome gene set enrichment. The “GSVA” package in R was used to perform GSVA enrichment analysis to obtain results on the differences in signaling pathways between different groups. The data sources were the “Hallmark” gene sets downloaded from the MSigDB database.

### Construction of the nomogram

A nomogram based on the hub genes was constructed using the “rms” package to achieve early prediction of illness development. A ROC curve was drawn to demonstrate the discriminative ability of the model. We generated a calibration curve to compare the nomogram-based forecasts with the actual outcomes to evaluate the precision of the prediction. Moreover, clinical impact curve analysis and decision curve analysis (DCA) were carried out to assess the clinical usefulness of the nomogram.

### Construction of the drug‒gene interaction network and molecular docking of active components

The search for targeted drugs for feature genes was conducted using the Drug‒Gene Interaction Database (DGIdb), which is accessible at http://dgidb.genome.wustl.edu/. Molecular docking, a prevalent technique in drug screening, is employed to predict the interaction between disease targets and drug ligands. Small-molecule compound 3D structures were downloaded from the PubChem database in mol2 format. High-resolution (less than 2.5 Å) 3D structures of the hub genes were sourced from the Protein Data Bank (PDB) at https://www.rcsb.org/and saved in PDB format. To identify effective receptor‒ligand binding sites, CB-Dock2 (https://cadd.labshare.cn/cb-dock2/) was utilized. This tool enhances blind docking methods by integrating cavity detection, docking, and homology modeling fitting (Liu et al., 2022b). The vina scores were utilized to compute the binding energies between receptor‒ligand molecules, where lower binding energies signify stronger interactions. Binding energies below −5 kcal/mol are indicative of favorable binding activity.

### Cell culture and model construction

The AC16 cells used in this study were derived from a cell bank (Procell, Wuhan, China). We cultured AC16 cells in DMEM (Gibco, MA, United States), which included 10% FBS and 1% penicillin/streptomycin (Solarbio, Beijing, China), at a constant temperature of 37°C and 5% CO_2_. AC16 cells were split into a control group (Con group) and an oxygen‒glucose deprivation (OGD) group. The AC16 cells in the OGD group were cultured in glucose-free medium in an anoxic atmosphere (95% N_2_ and 5% CO_2_) for 6 h according to the relevant experimental conditions.

### Quantitative reverse transcription‒polymerase chain reaction (RT‒qPCR) analysis

TRIzol kits (Invitrogen, United States) were used to extract RNA from AC16 cells, and a QuanTiect reprogramming kit (Qiagen, Germany) was used to reverse transcribe the RNA into cDNA. The amount of DNA present at each PCR cycle was measured using quantitative PCR (qPCR). SYBR-Green (Takara, Japan) was used for the quantification of real-time qPCR results. The levels of GAPDH were used to normalize the expression levels. The primer sequences are listed in Table 1. The 2^−ΔΔCT^ approach was used for data analysis.

**TABLE 1:** Primers were used for quantitative reverse transcription‒polymerase chain reaction (RT‒qPCR).

Gene	Primers	Sequence (5′–3′)
*BCL6*	Forward	TGTACACATCTCGGCTCAATTTG
	Reverse	ATCTCTGCTTCACTGGCCTTAAT
*CDA*	Forward	GCCAGTGACATGCAAGATGATTT
	Reverse	CCAGGGCTGTGAGACATCTTTAT
*IL1R2*	Forward	CACCTACGTCTGCACTACTAGAA
	Reverse	TTCACTCAGGTCAGGGCATACTA
*GAPDH*	Forward	GGAGTCCACTGGCGTCTTCA
	Reverse	GTCATGAGTCCTTCCACGATACC

### Statistical analysis

Perl (version 5.18.2) was used to organize the data, and R (version 4.1.2) was used to analyze the data. The Wilcoxon rank sum test or Student’s t-test was used to determine whether there were any significant differences between the two independent groups. Unless otherwise indicated, *P* < 0.05 was considered statistically significant.

## Results

### Landscape of immune infiltration in AMI

**FIGURE 2:**
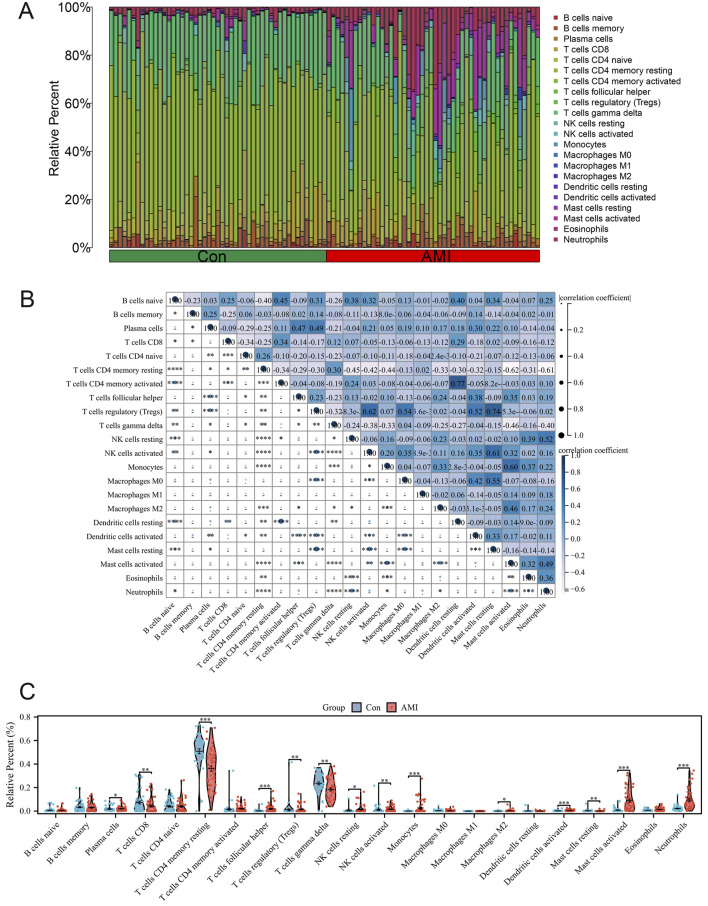
Landscape of immune infiltration in AMI. **(A)** Bar plot of the composition of different immune cells in each AMI and control sample. **(B)** Correlation analysis between different immune cells. **(C)** Violin plot of various immune cell proportions in the AMI and control groups.

Using the CIBERSORT algorithm, we assessed the immune-mediated infiltration levels of 22 immune cells in the two distinct sample groups. The differences between these immune cells are displayed in a stacked bar chart (Figure 2A). Immunocyte panoramic analysis showed a significantly higher ratio of macrophage/monocyte infiltration in AMI samples, marking the characteristics of an immune microenvironment dominated by myeloid inflammation. To determine the associations between immune cells, the correlation coefficients between two groups of immune cells were computed (Figure 2B). Significant positive correlations existed between neutrophils and resting NK cells, monocytes, and M2 macrophages. In addition, the AMI samples presented considerably increased relative infiltration of neutrophils, activated mast cells, and monocytes (Figure 2C). These results suggest that neutrophils might be crucial in AMI.

### Causal relationship between neutrophils and MI

**FIGURE 3:**
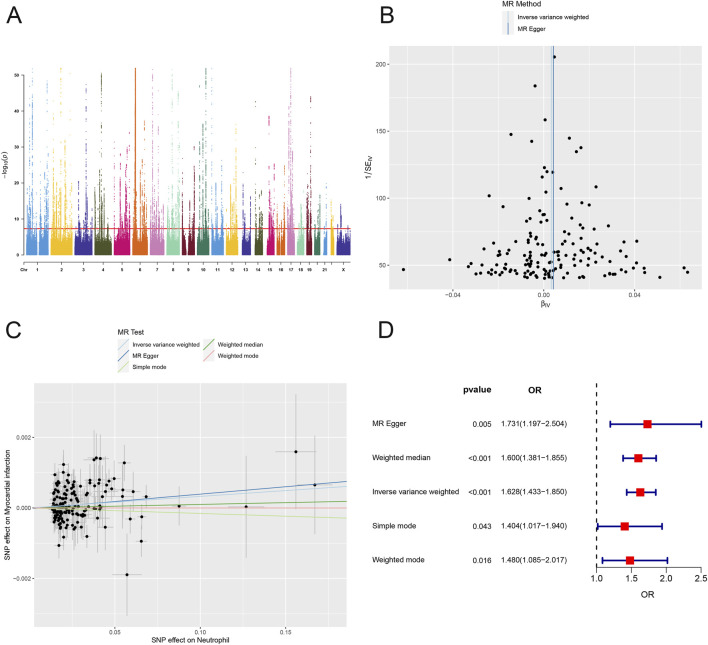
Causal relationship between neutrophils and AMI. **(A)** Rectangular Manhattan plot displaying information on neutrophil-associated SNP loci in the genome. **(B)** Funnel plot of instrumental variables, with points representing SNPs evenly distributed on both sides, indicating less heterogeneity. **(C)** Scatterplot of the causal effect of neutrophils on AMI levels. **(D)** Odds ratios (ORs) for estimates of the relationship between AMI and neutrophils. CI, confidence interval. The five lines represent MR Egger, weighted median, inverse variance weighted, simple mode, and weighted mode.

An estimation of the genome-wide genetic correlation between AMI and neutrophils revealed a strong signal on chromosome 6 (Figure 3A). SNPs with *P* < 5.0 × 10^−8^ were screened, linkage disequilibrium was removed, confounders were rejected via a PhenoScanner website search, weak variables with F statistics <10 and SNPs with palindromic structure were removed; in total, 162 SNPs were identified to evaluate the causal relationship between neutrophils and AMI (Supplementary Table S1). The effect of each SNP on the outcome is demonstrated in Supplementary Figure S1. Q tests for heterogeneity between the IVW and MR‒Egger methods were subsequently performed. We also constructed funnel plots of the causal relationship between neutrophils and AMI, and the funnel plots were symmetric, indicating that there was little heterogeneity in the results (Figure 3B). In leave-one-out analyses, after removing either SNP, the effect intervals of the remaining SNPs on the effect of the outcome were all to the right of the null line, which was similar to the overall effect interval, suggesting that a specific SNP did not drive the causal association; therefore, the MR results were stable (Supplementary Figure S2). The scatterplot shows the relationship between the effect of SNPs on outcomes and the impact of SNPs on exposure factors, with each method identifying neutrophils as a risk factor for AMI. The MR‒Egger intercept in the plot was nearly equal to 0, indicating the absence of horizontal pleiotropy (Figure 3C). IVW is the main sensitivity analysis method for MR, which calculates an estimate of the overall causal effect by weighing the effect of each genetic variant on the outcome. This finding demonstrated a statistically significant possible causative relationship between neutrophils and AMI risk (OR: 1.628; 95% CI: 1.433–1.850; *P* < 0.001). Other Mendelian analysis methods, including MR‒Egger, weighted median, simple mode, and weighted mode, were also conducted, and their results all showed the same effect as IVW (Figure 3D). These findings demonstrate that neutrophils and AMI continue to have a strong causal connection. The greater the SNP effect on neutrophils is, the greater the risk of AMI.

### Acquisition of neutrophil coexpressed genes

**FIGURE 4:**
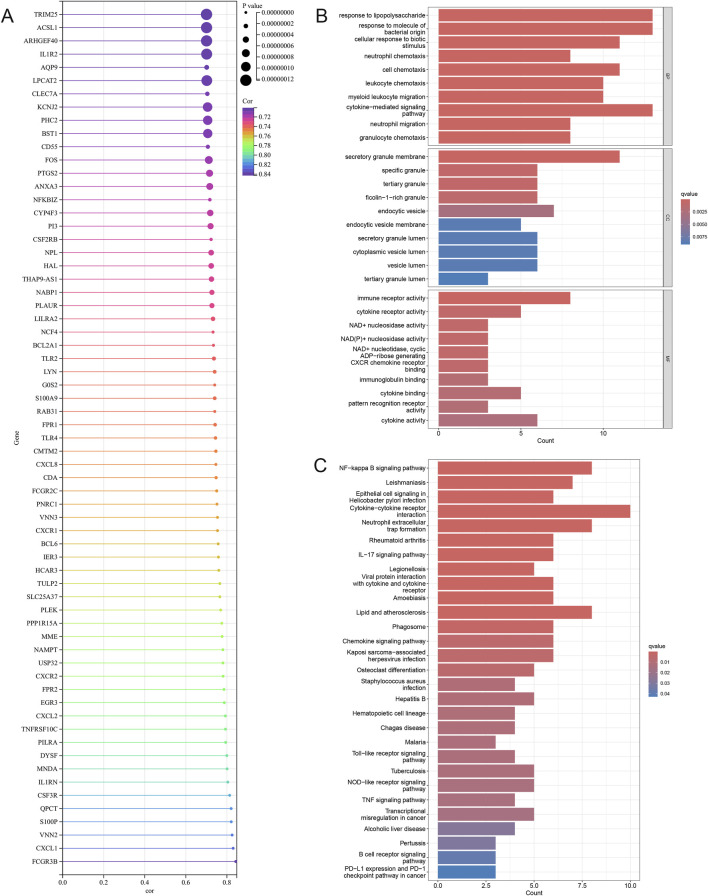
Acquisition and functional enrichment analysis of neutrophil coexpressed genes. **(A)** Bubble plot showing genes closely related to neutrophils. The color of the bubble represents the value of the correlation coefficient, and the size indicates the *P* value. **(B)** Bar plot of GO enrichment analysis of biological processes, molecular functions, and cellular components. **(C)** Bar plot showing the results of the KEGG enrichment analysis of neutrophil coexpressed genes.

The Pearson correlation analysis revealed the interaction network of neutrophils with multiple genes, and 65 genes with correlation coefficients >0.7 associated with the expression levels of neutrophils were identified (Figure 4A). We performed functional enrichment analysis to clarify the potential biological effects of these neutrophil coexpressed genes. The GO circle diagram shows that these genes were enriched mainly at the biological process level. Specifically, they were closely related to the cellular response and chemotaxis to various substances, among which the responses to lipopolysaccharide, a molecule of bacterial origin and biotic stimulus, and neutrophil, leukocyte, and granulocyte chemotaxis were significantly enriched; additionally, the cytokine-mediated signaling pathway should not be overlooked. Immune receptor activity was also related to molecular function, and the secretory granule membrane was a critical cell component (Figure 4B). The KEGG results suggested that the NF-κB and IL-17 signaling pathways were both important (Figure 4C). In addition, lipids and atherosclerosis showed relationships with these genes. These results suggest that neutrophil-associated gene clusters may drive AMI progression through a cascade axis of “chemotaxis - NF-κB/IL-17 inflammatory pathway activation - cardiomyocyte injury”, which provides novel therapeutic targets for targeting the neutrophil inflammatory response.

### Identification of AMI-related neutrophil coexpressed genes

**FIGURE 5:**
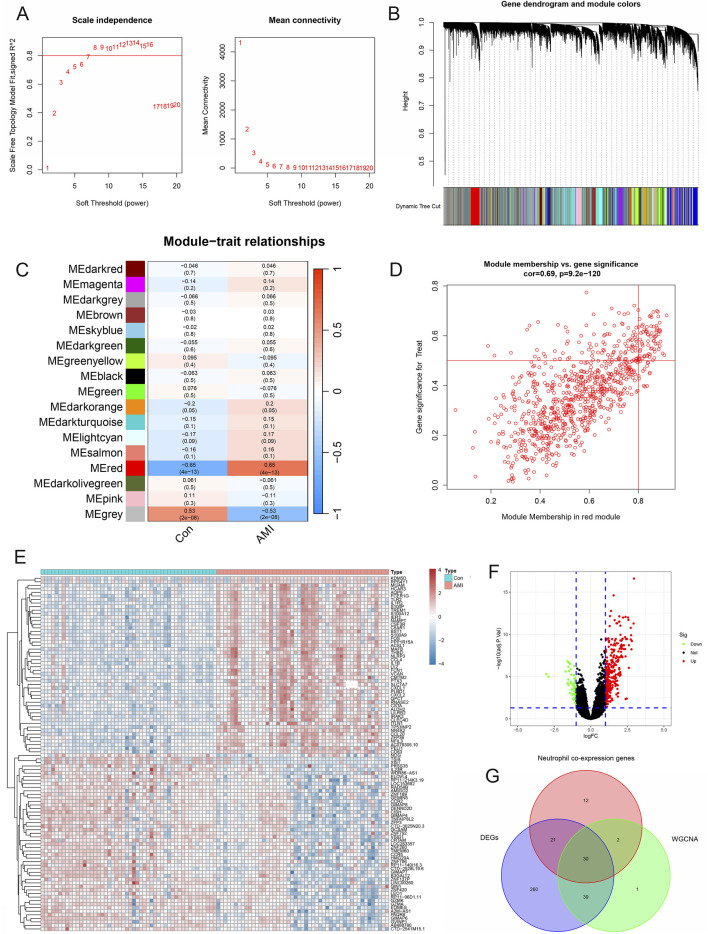
Identification of AMI-related neutrophil coexpressed genes. **(A)** Scale-free exponential analysis and average connectivity analysis of various soft thresholds. **(B)** Clustering dendrogram of all different genes. **(C)** Plot of module–trait relationships. Correlation coefficients and corresponding *P* values between genes and AMI are shown for each module. **(D)** Scatterplot of module membership vs gene significance in the red module. **(E)** Heatmap of differentially expressed genes in the AMI and control groups. **(F)** Volcano plot revealing the DEGs between the control and AMI groups. **(G)** Venn diagram of the intersection of neutrophil coexpressed genes, DEGs, and genes in the red module.

According to Figure 5A, when the scale-free fitting index was 0.8, the minimum soft threshold that met the construction of the scale-free network was 7. Similarly, the decrease in mean connectivity was reduced when the soft threshold was ≥7; therefore, we used it as the optimal soft threshold for the subsequent analysis. By merging modules with high similarity (Figure 5B) and visualizing the correlations between modules and traits (Figure 5C), 17 gene coexpression modules were obtained, from which the MEred module genes were the darkest in color, indicating the close and positive associations between these genes and AMI. In contrast, MEred module genes were inversely related to AMI. Correlation analysis between gene significance and module membership was subsequently performed, and the scatterplot revealed a positive association between genes in the red module and AMI, indicating that the red module was the most critical one on which to focus (Figure 5D). We analyzed the 350 DEGs and generated a volcano plot to show the up- and downregulated genes (Figures 5E,F). The intersection results of the neutrophil coexpressed genes, module genes, and DEGs were subsequently visualized as a Venn diagram, which revealed 30 AMI-related neutrophil coexpressed genes for further study (Figure 5G).

### Molecular typing based on AMI-related neutrophil coexpressed genes

**FIGURE 6:**
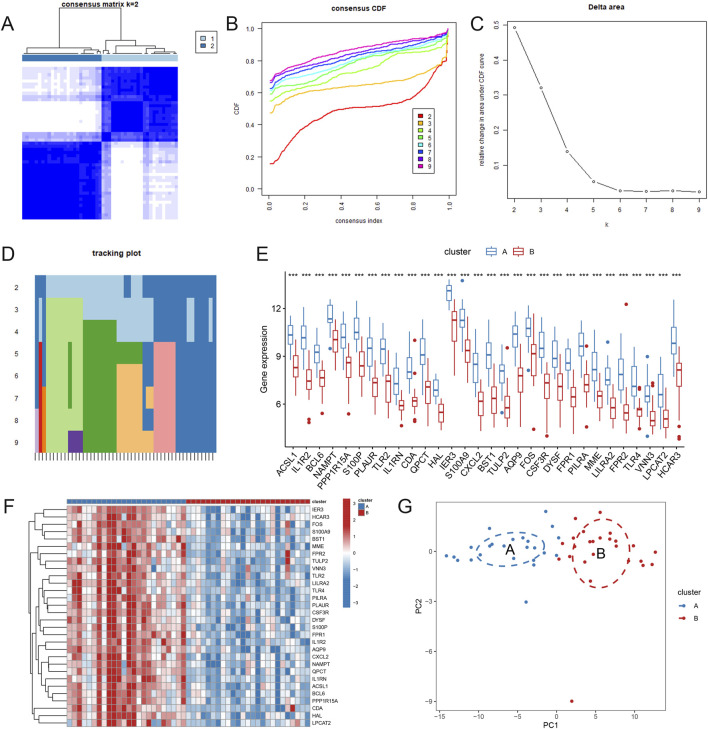
Molecular typing based on AMI-related neutrophil coexpressed genes. **(A)** Consensus matrix with k = 2. **(B)** Cumulative distribution function (CDF) when different k values are chosen. **(C)** Delta area plot of consensus clustering, indicating the relative changes in the area under the CDF curve. **(D)** Tracking plot demonstrating the change in sample classification. The columns represent the samples; the rows represent different k values. **(E)** Boxplots showing the expression levels of 30 AMI-related neutrophil coexpressed genes in different clusters. **(F)** Heatmap of the expression of 30 AMI-related neutrophil coexpressed genes in different clusters. **(G)** Principal component analysis (PCA) of subclusters A and **(B)** *P < 0.05; **P < 0.01; ***P < 0.001.

To determine the optimal number of clusters for the dataset, cluster-free analysis was performed with the “ConsensusClusterPlus” package, and k = 2 was used for the subsequent analysis (Figures 6A–D). In both neutrophil-related expression patterns, the expression levels of the 30 AMI-related neutrophil coexpressed genes were significantly greater in Cluster A than in Cluster B (Figures 6E,F). The PCA results indicated that the 30 AMI-related neutrophil coexpressed genes could effectively distinguish the two neutrophil-related expression patterns (Figure 6G). These results further confirm the accuracy of the determination of neutrophil coexpressed genes.

### Immune infiltration analysis of different molecular clusters

**FIGURE 7:**
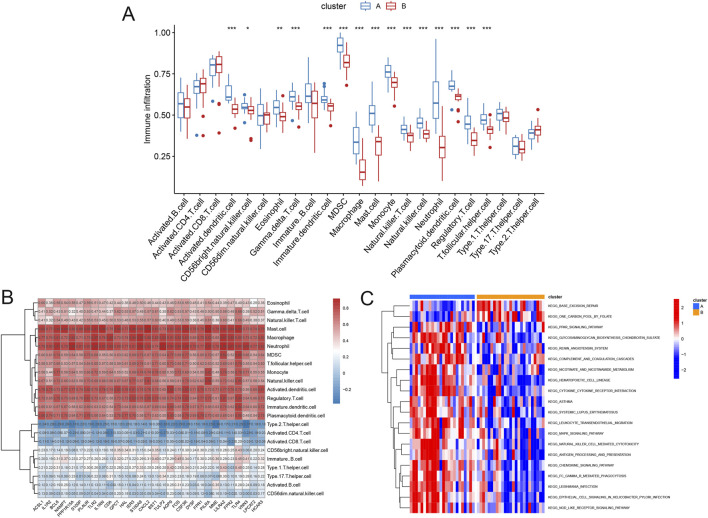
Immune infiltration analysis of different molecular clusters. **(A)** Boxplot revealing the infiltration conditions of diverse immune cells. **(B)** Heatmap illustrating the relationships between 30 AMI-related neutrophil coexpressed genes and various immune cells. **(C)** KEGG pathway analysis of the A and B clusters. *P < 0.05; **P < 0.01; ***P < 0.001.

Subsequently, immune infiltration analysis was performed using ssGSEA to investigate the relationships between different subpopulations and immune infiltration. In Cluster A, AMI-related neutrophil coexpressed genes, such as activated dendritic cells, macrophages, monocytes, and regulatory T cells, were significantly more common in most immune cells than in Cluster B (Figure 7A). To better understand the associations between the hub genes and immune cells, we plotted a heatmap to visualize them. The results revealed that AMI-related neutrophil coexpressed genes were significantly positively correlated with mast cells, macrophages, and neutrophils and significantly negatively correlated with type 2 helper T cells, activated CD4^+^ T cells, and activated CD8^+^ T cells (Figure 7B). Furthermore, GSVA was performed to explore the pathways associated with the two neutrophil-related expression patterns. Cluster A was significantly positively correlated with most KEGG pathways, including cytokine–receptor interaction, MARK, and NOD-like receptor signaling pathways. Moreover, base excision repair and one carbon pool by folate were more closely linked to Cluster B (Figure 7C). These results indicate that both high and low expression patterns of AMI-related neutrophil coexpressed genes reflect different levels of immune cell infiltration in AMI.

### Identification and validation of hub neutrophil coexpressed genes in AMI

**FIGURE 8:**
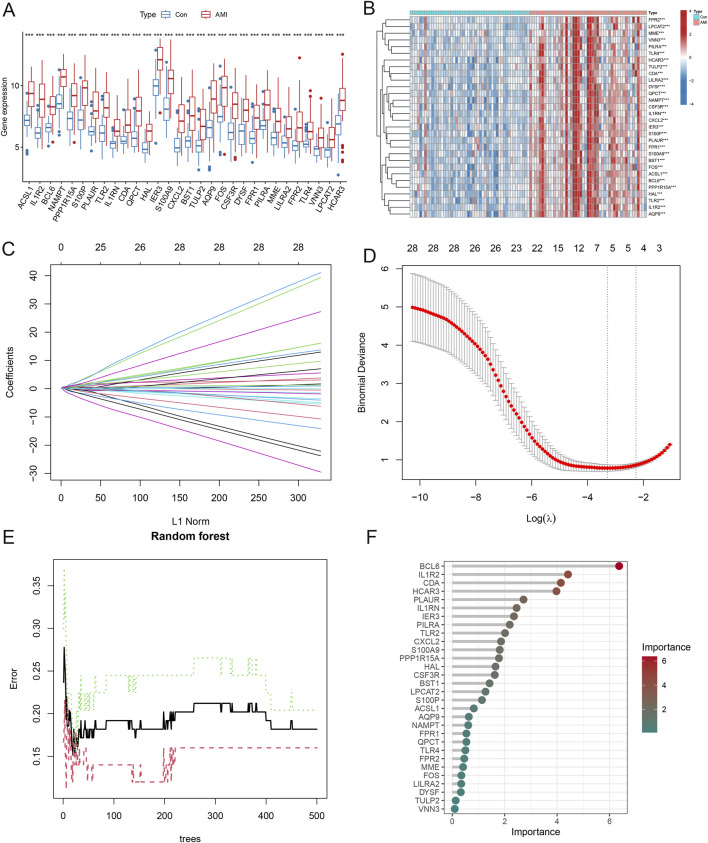
Identification of hub neutrophil coexpressed genes in AMI. **(A)** Boxplot demonstrating the expression levels of 30 candidate genes in different groups. **(B)** Heatmap showing the gene expression levels in the other groups. **(C)** LASSO regression analysis cross-validation curves indicating the convergence screening process for 30 genes. **(D)** Coefficient spectrum in the LASSO regression model. The vertical coordinate is binomial deviance, and the horizontal coordinate is the log(λ) value of the penalty coefficient. **(E)** The reverse cumulative distribution of residuals indicates the error rates when different numbers of trees are selected. **(F)** Importance scores for each gene. The X-axis represents scores based on importance, and the Y-axis represents different candidate genes. *P < 0.05; **P < 0.01; ***P < 0.001.

Differential analysis of these AMI-related neutrophil coexpressed genes revealed that all the genes were highly expressed in the AMI group (Figures 8A,B). The LASSO regression was used to screen the parameters, and the changes in the coefficients of these variables are characterized in Figure 8C. Applying the 10-fold cross-validation method to the iterative analysis, 6 genes (*ACSL1*, *IL1R2*, *BCL6*, *NAMPT*, *S100P*, and *CDA*) were ultimately extracted when λ was 0.037 (Logλ = −3.288) from the initial 30 candidate AMI-related neutrophil coexpressed genes (Figure 8D). RF feature selection shows that the model error enters a stable plateau period when ntree = 400 and the model stabilizes (Figure 8E). Figure 8F represented the importance score of each gene, 9 genes (*BCL6*, *IL1R2*, *CDA*, *HCAR3*, *PLAUR*, *IL1RN*, *IER3*, *PILRA*, and *TLR2*) were screened based on the criterion of MDG >2.

**FIGURE 9:**
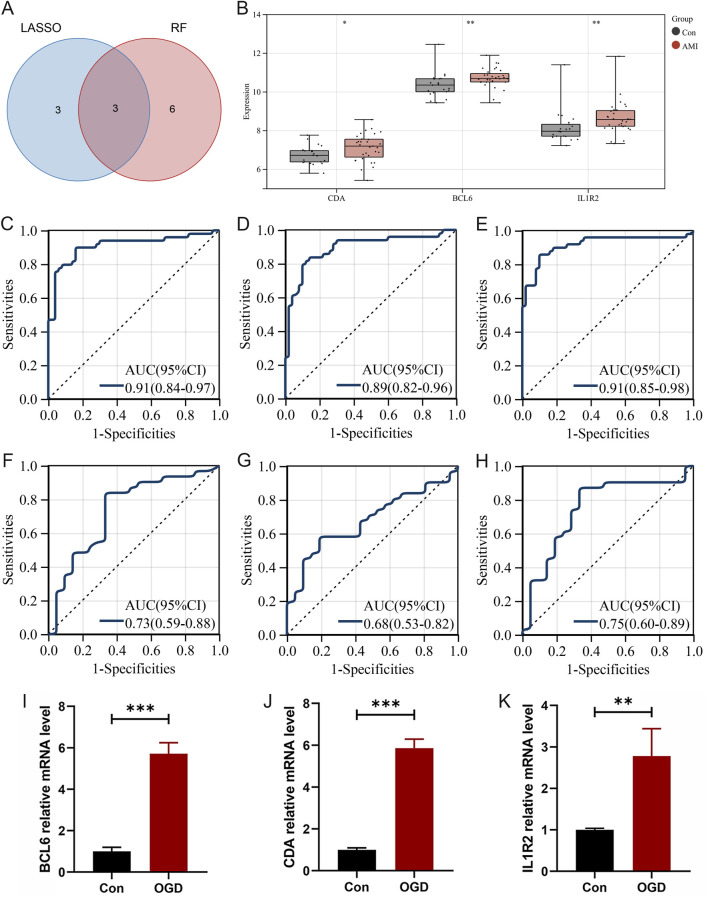
Validation of hub neutrophil coexpressed genes in AMI. **(A)** Venn diagram of the intersection of LASSO and RF analyses. **(B)** Boxplot displaying the expression levels of *CDA*, *BCL6*, and *IL1R*2 in GSE48060 dataset. **(C–E)** ROC curves for *BCL6*, *CDA*, and *IL1R2* separately in the training dataset. **(F–H)** ROC curves for *BCL6*, *CDA*, and *IL1R2* separately in the validation dataset. **(I–K)** RT‒qPCR to detect the expression levels of 3 hub neutrophil coexpressed genes in the Con and OGD samples (n = 4 independent experiments). OGD, oxygen‒glucose deprivation. *P < 0.05; **P < 0.01; ***P < 0.001 by Student’s t-test.

Combining the results of the two algorithms, we identified 3 hub neutrophil coexpressed genes in AMI: *BCL6*, *CDA*, and *IL1R2* (Figure 9A). The findings revealed substantial variation in the expression of the hub genes, all of which were increased in the AMI group compared with the control group based on the testing set (GSE48060 dataset) (Figure 9B). ROC curves were drawn for the training dataset, and all three genes had high diagnostic values, with AUCs of 0.91, 0.89, and 0.91, respectively (Figures 9C–E). In the testing dataset, the AUC of these genes was approximately 0.7, which verified the accuracy of the 3 hub genes (Figures 9F–H). To further confirm the accuracy of our findings, we created a cellular model of AMI by subjecting AC16 cells to glucose and oxygen deprivation. Compared with those in the control groups, the expression levels of *BCL6*, *CDA*, and *IL1R2* in the OGD group were significantly increased (Figures 9I–K). These findings further supported the results of the bioinformatics analysis.

### Biological functions of hub neutrophil coexpressed genes in AMI

**FIGURE 10:**
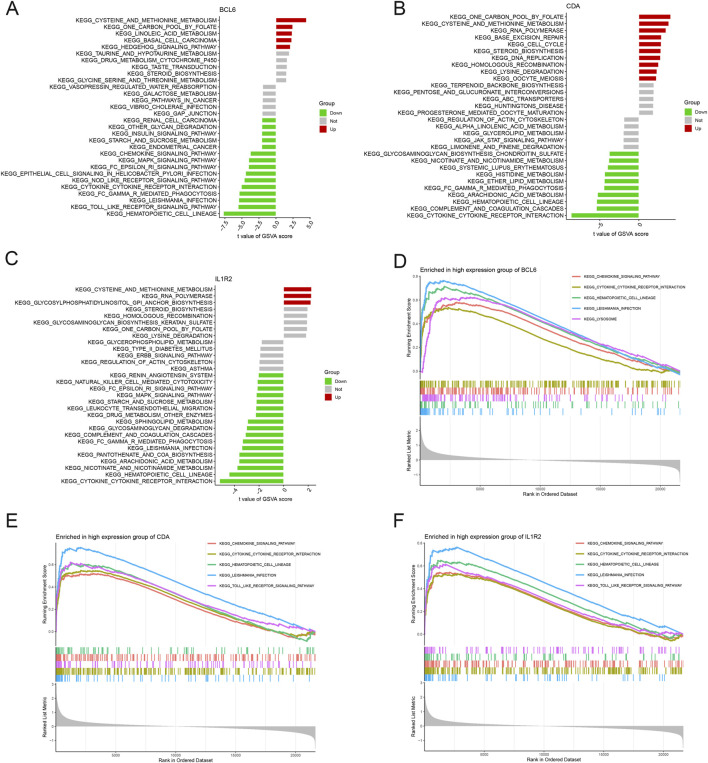
Biological functions of hub neutrophil coexpressed genes in AMI. **(A–C)** GSVA analysis of *BCL6, CDA*, and *IL1R2*. The horizontal axis represents the t value of the GSVA score; the Y-axis represents different KEGG function pathways. **(D–F)** GSEA of *BCL6*, *CDA*, and *IL1R2*. Each vertical line in the figure represents one gene in a specific KEGG pathway gene set, and the peaks appear on the left side, indicating that the core molecules in the gene set are mainly concentrated in the highly expressed group.

In GSVA, all genes related to cysteine and methionine metabolism were upregulated, whereas those related to cytokine‒receptor interactions and the hematopoietic cell lineage were downregulated. Moreover, *IL1R2* and *BCL6* were negatively related to Leishmania infection (Figures 10A–C).

The high-expression groups of *BCL6*, *CDA*, and *IL1R2* were enriched in Leishmania infection, hematopoietic cell lineage, cytokine receptor interaction, and chemokine signaling pathways (Figures 10D–F). Moreover, *IL1R2* and *CDA* were also associated with TLR signaling pathways. *BCL6* was correlated with lysosomes.

### Establishment of the nomogram

**FIGURE 11:**
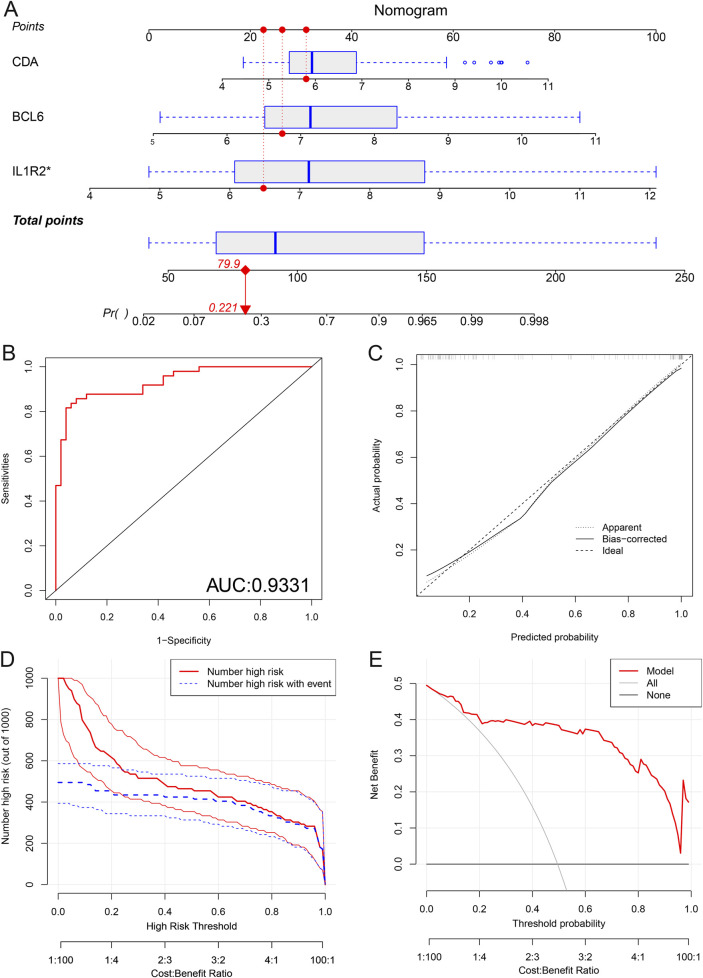
Construction of the nomogram. **(A)** The nomogram is based on 3 hub neutrophil coexpressed genes. The score for each factor is determined by projecting each variable upward to its corresponding point value. The risk score for developing AMI is obtained by adding the scores for each item. **(B)** ROC curve of the predicted model. **(C)** Calibration curve used to verify the accuracy of the model. **(D)** Clinical impact curve (CIC) constructed to predict the model with high precision. **(E)** Decision curve analysis (DCA). The horizontal coordinate represents the percentage of threshold probability, and the vertical axis represents the net benefit.

Based on how much each hub neutrophil coexpressed gene contributes to AMI, we constructed a nomogram to better diagnose AMI (Figure 11A). We plotted the ROC curve to assess the model’s discriminative ability, and the results showed excellent accuracy (AUC = 0.993) (Figure 11B). In the calibration curve (Figure 11C), the bias-corrected curve was close to the 45-degree diagonal, indicating that the model performed well. We used the model to predict the risk stratification of the population, comparing the high-risk population classified by the model with the actual situation (Figure 11D). To assess the nomogram’s net benefit, our model’s curve was away from the two extreme curves, illustrating its good application value (Figure 11E). All the results mentioned above indicate that the nomogram can accurately predict the incidence of AMI from different perspectives.

### Causal relationships between hub neutrophil coexpressed genes and MI

**FIGURE 12:**
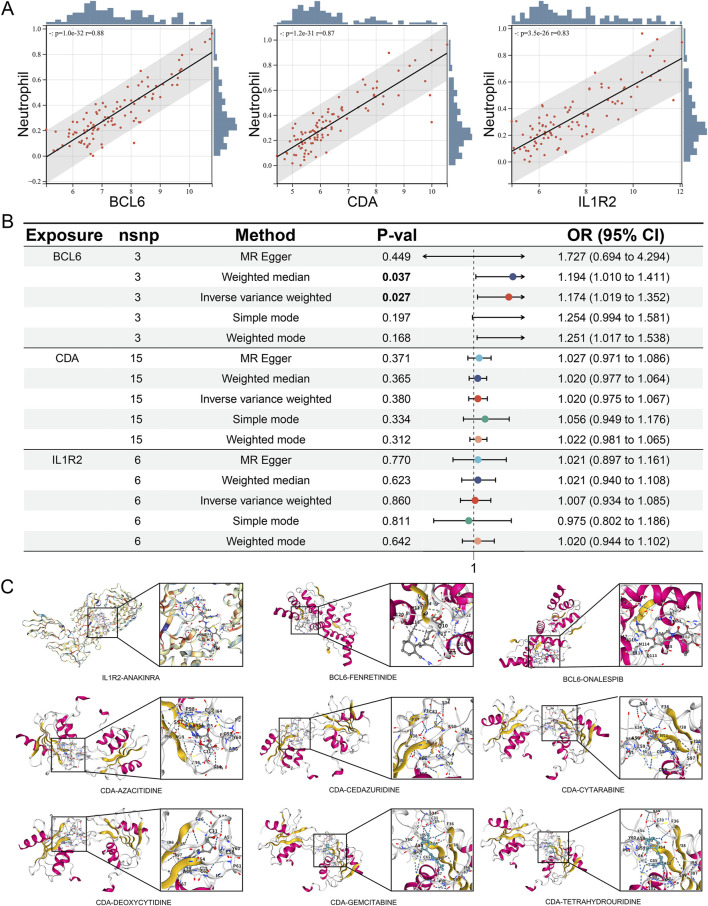
Verification of causality and molecular docking. **(A)** Pearson correlation analysis between 3 hub neutrophil coexpressed genes and neutrophil expression levels. **(B)** Forest plot of Mendelian randomization results for 3 hub neutrophil coexpressed genes and MI. **(C)** Results of the molecular docking of active compounds that target drugs and hub neutrophil coexpressed genes.

Figure 12A shows consistently high positive correlations between these 3 hub neutrophil coexpressed genes and AMI neutrophils (all |r| > 0.8, *P* < 0.05). To further explore the causal relationships between the 3 hub neutrophil coexpressed genes and MI, we ultimately identified 26,152 SNPs as IVs, all of which satisfied the three fundamental assumptions of MR. Each of the selected SNPs presented F statistics greater than 10 (Supplementary Table S2). The associations of 3 independent *BCL6* genetic IVs, 15 independent *CDA* genetic IVs, and 6 independent *IL1R2* genetic IVs in the MI GWAS are shown in Table 2. The findings demonstrated a substantial positive causal association between MI and *BCL6* (OR: 1.174, 95% CI: 1.019–1.352; *P* = 0.027) in the MR analysis using the inverse variance weighted technique (Figure 12B). For additional validation, the weighted mode, weighted median, and simple mode were employed in addition to MR‒Egger. For *BCL6*, the weighted median and IVW consistently increased the risk of MI (OR > 1, *P* < 0.05) (Figure 12B). Table 3 presents in detail the MR analysis results of the causal associations between genes and MI. The impact of heterogeneity and pleiotropy on the results did not need to be considered, as this was supported by the pleiotropy and heterogeneity tests for the coexpressed genes, which yielded *P* values >0.05 (Table 4). The sensitivity analysis demonstrated the robustness of the analysis, which revealed that the effect sizes of the included IVs were similar to the total effect size (Supplementary Figure S3).

**TABLE 2:** Association of genetic instrumental variants (IVs) with myocardial infarction (MI).

Exposure	SNP	Exposure			Outcome		
		Beta	SE	*P* value	Beta	SE	*P* value
*BCL6*	rs12630483	0.12165900	0.01661070	0.00000000	0.02790000	0.02380000	0.24050000
	rs3172469	0.17040800	0.01340100	0.00000000	0.03790000	0.01700000	0.02565020
	rs7210990	0.12557000	0.01186430	0.00000000	0.00450000	0.01560000	0.77210100
*CDA*	rs115601582	1.52913000	0.02338110	0.00000000	0.02150000	0.03600000	0.55010000
	rs12264390	−0.08083920	0.01456250	0.00000003	−0.01400000	0.01930000	0.46710000
	rs141856780	−0.28531600	0.04623880	0.00000000	0.03530000	0.06650000	0.59560000
	rs144719866	−0.29706500	0.04295140	0.00000000	0.09090000	0.05660000	0.10830000
	rs147202062	−0.28409300	0.03389570	0.00000000	−0.00990000	0.03670000	0.78679900
	rs148645028	−0.37608700	0.04535650	0.00000000	0.16430000	0.07430000	0.02698980
	rs149007767	−0.15910600	0.01662780	0.00000000	0.01010000	0.01950000	0.60450000
	rs149110519	0.25127600	0.02657370	0.00000000	−0.00630000	0.04870000	0.89790000
	rs150302054	−0.34629000	0.04620030	0.00000000	0.06680000	0.06720000	0.31990000
	rs2298295	1.08051000	0.03500400	0.00000000	0.04180000	0.06690000	0.53260000
	rs55738402	−0.28454100	0.04479630	0.00000000	−0.05690000	0.07910000	0.47140000
	rs56330463	−0.08524780	0.01204590	0.00000000	−0.01530000	0.01610000	0.34450000
	rs78865488	0.41678800	0.02963950	0.00000000	0.08420000	0.03310000	0.01094990
	rs80074940	1.28616000	0.04795250	0.00000000	0.09390000	0.09660000	0.33080000
	rs8078723	−0.06700150	0.01224940	0.00000005	0.02330000	0.01590000	0.14300000
*IL1R2*	rs28498283	0.08140050	0.01365000	0.00000000	−0.02700000	0.01780000	0.12970000
	rs35979828	−0.19233800	0.02559800	0.00000000	0.03720000	0.05210000	0.47570000
	rs56330463	−0.07512040	0.01205070	0.00000000	−0.01530000	0.01610000	0.34450000
	rs62155149	0.37520700	0.01227230	0.00000000	0.00770000	0.01580000	0.62550000
	rs7210990	0.09950380	0.01188180	0.00000000	0.00450000	0.01560000	0.77210100
	rs78423067	−0.28050000	0.04996880	0.00000002	0.03560000	0.05230000	0.49630000

**TABLE 3:** The causal association of hub genes with myocardial infarction (MI).

Exposure	Method	nSNP	Beta	Beta_lci95	Beta_uci95	SE	*P* value	OR	OR_lci95	OR_uci95
*BCL6*	MR Egger	3	0.54629791	−0.36465880	1.45725461	0.46477383	0.44877930	1.72684822	0.69443355	4.29415421
	Weighted median	3	0.17724827	0.01733976	0.33715678	0.08158598	0.02981544	1.19392747	1.01749096	1.40095869
	IVW	3	0.16019578	0.01852434	0.30186722	0.07228135	0.02667226	1.17374065	1.01869698	1.35238165
	Simple mode	3	0.22595872	−0.00860097	0.46051842	0.11967331	0.19961690	1.25352392	0.99143591	1.58489541
	Weighted mode	3	0.22363015	0.02274911	0.42451119	0.10249033	0.16084315	1.25060840	1.02300985	1.52884293
*CDA*	MR Egger	15	0.02647656	−0.02952983	0.08248294	0.02857469	0.37103062	1.02683017	0.97090192	1.08598015
	Weighted median	15	0.01968961	−0.02223715	0.06161636	0.02139120	0.35733579	1.01988473	0.97800828	1.06355425
	IVW	15	0.02019028	−0.02492882	0.06530939	0.02301995	0.38044448	1.02039549	0.97537934	1.06748925
	Simple mode	15	0.05477932	−0.05927609	0.16883474	0.05819154	0.36247997	1.05630749	0.94244653	1.18392447
	Weighted mode	15	0.02209861	−0.01914755	0.06334476	0.02104396	0.31145452	1.02234459	0.98103460	1.06539408
*IL1R2*	MR Egger	6	0.02062070	−0.10846431	0.14970570	0.06585970	0.76985036	1.02083477	0.89721092	1.16149237
	Weighted median	6	0.02053898	−0.06056658	0.10164454	0.04138039	0.61965048	1.02075136	0.94123110	1.10698991
	IVW	6	0.00670395	−0.06792849	0.08133640	0.03807778	0.86024723	1.00672647	0.93432728	1.08473574
	Simple mode	6	−0.02516812	−0.21676433	0.16642810	0.09775317	0.80707931	0.97514596	0.80511969	1.18107861
	Weighted mode	6	0.01955905	−0.06373598	0.10285409	0.04249747	0.66466589	1.01975158	0.93825268	1.10832968

**TABLE 4:** Pleiotropy and heterogeneity test of exposure instrumental variants (IVs) in myocardial infarction (MI).

Exposure	Pleiotropy test			Heterogeneity test					
	MR egger			MR egger			IVW		
	Intercept	SE	*P* value	Q	Q-df	Q-*P* value	Q	Q-df	Q-*P* value
*BCL6*	−0.05596815	0.06655243	0.55485994	0.80858006	1.00000000	0.36854039	1.51579940	2.00000000	0.46864970
*CDA*	−0.00447433	0.01131270	0.69887767	20.00096373	13.00000000	0.09518623	20.24163884	14.00000000	0.12270532
*IL1R2*	−0.00364678	0.01373271	0.80370706	4.38941572	4.00000000	0.35586202	4.46680020	5.00000000	0.48433771

### Drug–hub gene interactions

By exploring the drug‒gene interactions in the DGIdb database for 3 hub neutrophil coexpressed genes, we identified and confirmed 12 potential drugs for treating AMI (Table 5).

**TABLE 5:** Candidate drugs targeting 3 hub neutrophil coexpressed genes.

Number	Gene	Drug	Interaction types	Interaction score
1	*CDA*	CYTARABINE	antineoplastic agent	0.373
2	*CDA*	GEMCITAB1NE	antineoplastic agent	0.162
3	*CDA*	TETRAHYDROURIDINE	-	11.187
4	*CDA*	DAUNORUBICIN LIPOSOMAL	antineoplastic agent	0.164
5	*CDA*	CEDAZURIDINE	-	3.729
6	*CDA*	DEOXYCYTIDINE	-	3.729
7	*CDA*	AZACITIDINE	antineoplastic agent	0.373
8	*BCL6*	FENRETINIDE	antineoplastic agent	1.289
9	*BCL6*	ONALESPIB	antineoplastic agent	4.350
10	*BCL6*	POLATUZUMAB VEDOTIN	-	0.916
11	*IL1R2*	ANAKINRA	DMARD, anti-inflammatory agent	5.800
12	*IL1R2*	RECOMBINANT INTERLEUKIN-1	antineoplastic agent	1.450

### Molecular docking verification of active compounds that target drugs and hub neutrophil coexpressed genes

We selected compounds corresponding to three key targets for molecular docking, with the aim of assessing the binding efficacy between the drug’s active compounds and the disease targets. No record of strict matching between recombinant interleukin-1 and daunorubicin liposomal was identified in the PubChem database. In addition, molecular docking could not be completed for polatuzumab vedotin because of its lower docking accuracy with *BCL6*. The results presented in Table 6 demonstrate that the binding energies ranged between −8.4 and −7.4 kcal/mol, indicating excellent binding activity between the drugs and the three key targets (Figure 12C).

**TABLE 6:** Molecular docking results of 3 hub neutrophil coexpressed genes.

Targets	PDB ID	Compounds	Cavity volume (Å3)	Affinity (kCal/mol)	Docking size			Center		
					x	y	z	x	y	z
*BCL6*	1R28	ONALESPIB	421	−7.7	25	25	25	−9	−14	−9
		FENRETINIDE	273	−7.4	28	28	28	−9	−4	−16
*IL1R2*	6U6U	ANAKINRA	2,374	−7.8	25	31	25	4	18	11
*CDA*	1MQ0	AZACITIDINE	222	−7.6	19	19	19	37	85	119
		CEDAZURIDINE	224	−7.8	18	18	18	33	93	106
		CYTARABINE	222	−8	19	19	19	37	85	119
		DEOXYCYTIDINE	224	−8	19	19	19	33	93	106
		GEMCITAB1NE	224	−8.4	19	19	19	33	93	106
		TETRAHYDROURIDINE	222	−7.9	19	19	19	37	85	119

## Discussion

After ischemic injury occurs in the myocardium, numerous neutrophils are recruited to and activated in the infarcted area, which is closely related to many pathological processes and mechanisms of AMI. The role of neutrophils in AMI has long attracted the attention of scholars. However, the precise cause‒and‒effect link between neutrophils and AMI remains to be determined because other factors and costs have hampered previous animal and observational studies. In this study, using two-sample MR, we identified neutrophils as a risk factor for AMI and confirmed the causal connection between neutrophils and AMI. In addition, we identified 3 genes that characterize neutrophils in AMI and constructed a nomogram that predicts AMI disease risk. We also systematically investigated the general biological features of AMI and the immune cell infiltration patterns in AMI under varying neutrophil-associated gene expression profiles, which can contribute to guiding clinical immunotherapy regimens more effectively. Finally, eQTL analysis revealed genetic variation in the expression of regulatory genes in AMI, which identified *BCL6* as a risk gene for AMI.

First, the study revealed a significant causal relationship between neutrophils and AMI by MR and AMI immune analyses; the greater the SNP effect on neutrophils was, the greater the risk of AMI. The pathogenesis of AMI is mediated by systemic, intraplaque, and inflammatory factors. Studies have shown that neutrophils are strongly associated with all culprit lesions in patients who die from AMI (Carbone et al., 2013; Franck et al., 2017). Neutrophil infiltration of the infarcted myocardium mediates myocardial tissue injury through the release of ROS and protein hydrolases, including several proinflammatory alarm elements (such as S100A8/A9) and proteases through degranulation and NET formation (Luo et al., 2023). Excessive release of ROS mediates the formation of oxidized low density lipoprotein (LDL) and leads to matrix metalloproteinase activation, resulting in plaque rupture (Forrester et al., 2018); S100A8/A9 directly inhibits mitochondrial function under hypoxic conditions, causing cardiomyocyte death (Volz et al., 2012); myeloperoxidase (MPO) takes part in the oxidation of LDL, thereby accelerating foam cell formation and indirectly inducing endothelial dysfunction by interfering with nitric oxide metabolism (Zheng et al., 2004); and NETs prompt macrophages to secrete IL-1β and IL-18 via NLRP3 inflammatory vesicles, which in turn prompts NET secretion with a positive feedback effect (Langseth et al., 2020). Following AMI, the inflammatory environment of neutrophil infiltration favors plaque instability and rupture, leading to the death of vascular smooth muscle cells (Jiang et al., 2022). Several studies from various large cohorts conducted worldwide have also demonstrated that a high neutrophil count is associated with a greater risk of AMI. In a CALIBER cohort study, which included approximately 4% of the United Kingdom population, higher neutrophil counts strongly correlated with myocardial infarction outcomes (Shah et al., 2017). Another cohort study revealed that an increased neutrophil count is associated with impaired microvascular reperfusion and poor functional recovery following myocardial infarction, serving as an independent predictor of severe microvascular injury (Takahashi et al., 2007). However, the causal relationship between neutrophils and AMI has not been clarified owing to heterogeneity between study populations, disease states, and different confounders adjusted in the analytical models. This study explored the relationship between neutrophils and AMI, effectively reducing bias in clinical and experimental studies and elucidating the causality between the two. The strong association is mainly characterized by the proinflammatory role of neutrophils (as a risk factor). Existing studies have demonstrated that over-activated neutrophil signaling after AMI exacerbates cardiomyocyte apoptosis and adverse cardiac remodeling, whereas well-timed apoptosis of neutrophils at the site of injury promotes macrophage polarization toward a pro-resolving phenotype, thereby limiting inflammatory injury (Kim et al., 2024). Furthermore, although pro-inflammatory N1 neutrophils were consistently predominant after myocardial infarction (>80% at all time points), the proportion of reparative N2 neutrophils increased time-dependently (from 2.4% ± 0.6% at day 1 to 18.1% ± 3.0% at day 7) (Ma et al., 2016). These results suggest that modulating neutrophil fate to regulate inflammatory and repair processes may be an effective strategy for treating myocardial infarction. However, it is still difficult to precisely analyze the spatiotemporal dynamics of neutrophils after myocardial infarction, and the regulatory mechanisms of their subpopulation transition, functional heterogeneity, and their specific contributions to myocardial repair need to be explored in depth. Our study revealed a strong correlation between neutrophils and AMI. More studies are needed to determine the cutoff point of neutrophils in AMI and the anti-inflammatory treatment options for patients with AMI.

To further understand the functions of neutrophil-related genes and related pathways, we functionally enriched and typed differential genes in AMI neutrophils. GO analysis revealed that the secretory granule membrane is also an important cell component. Neutrophil granules contain proinflammatory factors, such as complement proteins, MPO, and neutrophil elastase (NE), which promote necrotic myocyte degradation in AMI. Neutrophil granules containing proinflammatory factors promote oxidative stress, which supports proteolytic catabolism of necrotic myocytes and the extracellular matrix in the context of AMI (Daseke et al., 2021). Secretory vesicles, one of the granule types, have several membrane-bound receptors, including complement receptor 1 and TLRs, which increase the ability of neutrophils to respond to inflammatory stimuli during granule–membrane binding (Lu et al., 2014). Therefore, understanding the granule secretion pathway and associated signaling mechanisms in neutrophils may contribute to intervention after myocardial infarction.

The KEGG results suggest that the NF-κB and IL-17 signaling pathways are essential pathways. NF-κB is a vital transcription factor that is mediated by TLRs and regulates innate immune and inflammatory responses, and gene expression profiles related to cell growth, cell survival, and cell death (Medzhitov et al., 1997; Zhang and Ghosh, 2001). Lu et al. discovered that TLR3 significantly contributes to myocardial injury in AMI by regulating nuclear translocation and binding activity, which promotes the inflammatory response and infiltration of inflammatory cells into the myocardium in AMI and ischemia/reperfusion (I/R) injury through the establishment of both mouse AMI and I/R models (Lu et al., 2014). Another study revealed that amelioration of oxidative stress and inflammation through modulation of the TLR4/MAPK/NF-κB pathway was cardioprotective against isoproterenol-induced AMI (Mi et al., 2023), which suggests that the NF-kB-related signaling pathway holds promise as an essential target for the treatment of AMI. Numerous studies have demonstrated that IL-17 is a cytokine with a potential role in AMI and participates in injury to or scarring of myocardial tissues, mainly by influencing the production of different inflammatory mediators in several types of cells and promoting the immune response (Mora-Ruíz et al., 2019). Zhou et al. reported that elevated IL-17 levels after AMI increased the infarct size, promoted myocardial fibrosis and apoptosis, and worsened cardiac function and poor prognosis (Zhou et al., 2014). A previous report revealed that IL-17 plays a pathogenic role in I/R injury by inducing neutrophil infiltration (Zhu et al., 2011), and we hypothesized that the IL-17 signaling pathway could mediate neutrophil infiltration of myocardial tissues and participate in the pathophysiological process of myocardial injury after AMI.

This study identified three hub genes (*BCL6*, *CDA*, and *IL1R2*) by LASSO regression and RF. *IL1R2*, a member of the IL-1 receptor family, functions as a key mediator of numerous cytokines triggered by immune and inflammatory responses. It plays a crucial role in regulating cellular metabolism and modulating cytokine-induced immune responses (Smith et al., 2004). Several studies have demonstrated the critical role of IL1R2 signaling in the inflammatory process of cardiovascular disease, and Orrem et al. reported that *IL1R2*, which is mediated by miR-383-3p, inhibits the activation of the inflammatory vesicle signaling pathway, which prevents apoptosis and inflammatory injury in coronary artery endothelial cells (Orrem et al., 2018). Another study reported a significant association between *IL1R2* and left ventricular remodeling in patients with ST-segment elevation myocardial infarction (Hanada and Kuramitsu, 1988). These findings suggest that *IL1R2* is significantly involved in the pathophysiologic process of AMI. The *CDA* gene encodes cytidine deaminase, which is involved in the metabolism of the pyrimidine analog gemcitabine. *CDA* has been used in the past, mainly for the treatment of biliary tract cancer, breast cancer, and other tumors (Iyer et al., 2013). Some current studies have also shown that *CDA* has high efficacy in treating acute myeloid leukemia, mainly through the PI3K/Akt pathway, which regulates apoptosis and thus influences the disease’s pathological process (Wei et al., 2018). Previous studies have demonstrated that the PI3K/AKT pathway plays a crucial role in AMI by regulating cardiomyocyte activity, angiogenic processes, and inflammatory responses (Ghafouri-Fard et al., 2022). Accordingly, *CDA* may regulate AMI cardiomyocytes and the process of development through PI3K/Akt; however, further experimental evidence is needed in the future.

*BCL6* is a sequence-specific DNA-binding protein that represses transcription by interacting with corepressor proteins, playing a crucial role in the cardiovascular system (Cardenas et al., 2017). One study revealed that *BCL6* synergizes with PPARD to protect the heart and inhibits adriamycin-induced sensitivity in cardiomyocytes (Altieri et al., 2012). Liu et al. reported that *BCL6*-carrying extracellular vesicles regulate cardiomyocyte hypoxic injury and apoptosis, inhibiting inflammation and cardiac remodeling after AMI through the BCL6/MD2/NF-κB axis (Liu et al., 2022a). Employing a bioinformatics approach, Zhou et al. reported that *BCL6* was significantly and positively correlated with upregulated neutrophils in AMI, which suggests that AMI is a possible future treatment target. (Zhou et al., 2022). In our current in-depth and meticulous research, we employed eQTL analysis, which not only strengthened the existing evidence of genetic associations but also further confirmed that *BCL6* is a crucial risk gene that plays a vital role in AMI pathogenesis. As a known transcriptional regulator, variations in *BCL6* expression levels may impact the homeostasis and function of cardiac tissue through complex regulatory networks, thereby increasing an individual’s risk of developing AMI. This discovery not only deepens our understanding of the genetic basis of AMI but also lays a solid theoretical foundation for the future development of novel therapeutic strategies targeting the *BCL6* pathway, with the aim of reducing the incidence of AMI and improving patient prognosis. All the above data indicate the role that *BCL6* plays in aging or cardiovascular disease and shows promise as an AMI diagnostic marker.

The GSEA results revealed that *BCL6*, *IL1R2*, and *CDA* were enriched in the chemokine signaling pathway. *IL1R2* and *CDA* are also associated with the TLR signaling pathway, whereas *BCL6* is associated with lysosomes. After AMI, the injured cardiomyocytes and matrix release of DAMPs, which mediate TLR signaling and ROS production in cardiomyocytes, induce the upregulation of cytokines and chemokines, which interact with the corresponding cytokine or chemokine receptors to recruit leukocyte subpopulations (Frangogiannis, 2014). Neutrophils are recruited in the region of myocardial infarction via chemokine-dependent and chemokine-independent pathways, causing vascular occlusion, the release of degradative enzymes, and the acceleration of ROS-induced cardiomyocyte death (Chao, 2009). AMI caused RAB7-mediated accumulation of aberrant mitochondrial–lysosomal contacts, which induced lysosomal enlargement and binding to autophagosomes and ultimately caused cardiac dysfunction (Yu et al., 2020). It suggests new targets for treating AMI. However, more in-depth studies should be conducted to understand the role of the lysosomal pathway in other pathologic conditions to identify additional targets for treating AMI disease.

By systematically quantifying the expression profiles of 3 hub neutrophil coexpressed genes, this study constructed a diagnostic model for AMI with excellent diagnostic efficacy (AUC = 0.933). The nomogram developed based on this model transforms the complex algorithm into a visualization tool and outputs individualized risk assessment results to support clinical decision-making. Future multicenter prospective studies are needed to validate the model’s ability to predict disease risk and hard clinical endpoints (e.g., 30-day cardiac mortality), and to assess its generalizability to real-world healthcare scenarios. In addition, targeting the 3 hub neutrophil coexpressed genes, we screened multiple potential candidates through targeted drug prediction to provide direction for subsequent intervention studies. Previous studies have confirmed that IL-1 is a key mediator of the inflammatory response in acute coronary syndrome (ACS), and IL-1 blockade with anakinra significantly inhibited CRP levels (at least partially) (Abbate et al., 2010; Abbate et al., 2013; Morton et al., 2015). However, the clinical benefit of IL-1 blockade in ACS remains controversial: the persistence of its efficacy and the risk of CRP rebound after discontinuation have not been clarified (Van Tassell et al., 2018). Of greater concern is that research on drugs targeting immune pathways in the cardiovascular field is still at an early stage. Although some clinical trials have shown their potential effectiveness, disease heterogeneity may lead to real-world efficacy differences, and future clinical translation will require large-scale studies to validate their safety and generalizability.

This study investigated the causal link between neutrophils and AMI using MR analysis. Furthermore, we conducted a comprehensive analysis of AMI-related neutrophil coexpressed genes in AMI diagnosis and immune infiltration based on bulk RNA sequencing data, offering a fresh perspective for future research. However, several limitations should be noted. First, the study was constrained by the limited number of samples sourced from a single database; genetic variations across different datasets could impact the accuracy of the statistical analysis. Second, potential discrepancies between datasets may introduce errors in our statistical findings. Although merging similar high-throughput sequencing data is a feasible option, it could exacerbate batch effects and distort the results. Third, due to incomplete patient information in public databases, we were unable to assess the model’s effectiveness in terms of disease progression and severity. In addition, to date, there is a lack of quantitative data on neutrophil counts and expression levels of *BCL6*, *CDA* and *IL1R2* genes in patients with AMI, which has somewhat limited depth of research and comprehensive evaluation. Consequently, further validation of our conclusions will require clinical cohort studies. Fourth, in the present study, only *in vitro* validation of cell lines was performed, which failed to provide a more comprehensive validation under the complex pathophysiological mechanisms embedded in the disease model. It is necessary to carry out *in vivo* experiments in animals that are more closely related to the pathological changes of the disease and even advance to human validation studies. Fifth, it is crucial to acknowledge that immune system dynamics play pivotal roles in pathologic changes during various stages of AMI. Single-cell sequencing across different immune backgrounds could provide insights into the dynamics of cardiac immunity following AMI. Sixth, in view of the lack of sufficient experimental data in the field of drug-gene interactions and molecular docking, further validation in cellular and *in vivo* and *ex vivo* models should be emphasized to provide a solid basis for in-depth exploration in this field. Seventh, this study was based on genetic data from a European population, and the applicability of the causal association between neutrophil levels, *BCL6* expression, and AMI in other populations still needs to be validated. Finally, our research confirms the essential role of *BCL6* as a key risk gene in neutrophil-mediated damage in AMI. However, the precise mechanisms underlying the immunoregulatory functions of *BCL6* in AMI remain unclear and warrant further experimental validation to elucidate their roles in this context.

## Conclusion

In conclusion, immune infiltration and MR analysis identified neutrophils as critical regulatory cells in AMI, and their causal relationship was verified. Two neutrophil-related expression patterns were effectively distinguished by molecular typing. Additionally, we identified 3 hub neutrophil coexpressed genes (*BCL6*, *IL1R2*, and *CDA*) associated with AMI. eQTL analysis further confirmed that *BCL6* plays a pivotal role as a key risk gene in neutrophil-mediated damage in AMI. However, more comprehensive studies are needed to determine the molecular mechanisms of AMI-related neutrophil coexpressed genes.

## Data Availability

The datasets presented in this study can be found in online repositories. The names of the repository/repositories and accession number(s) can be found in the article/Supplementary Material.
