# Supplementary material for: Identification and verification of biomarkers associated with neutrophils in acute myocardial infarction: integrated analysis of bulk RNA-seq, expression quantitative trait loci, and mendelian randomization
Source: Front Mol Biosci. 2025 Aug 8;12:1614350. doi: 10.3389/fmolb.2025.1614350 (PMC12371120; doi:10.3389/fmolb.2025.1614350)
Supplement: Supplementary file 2 [file DataSheet1.pdf]

Appendix A. Supplementary data

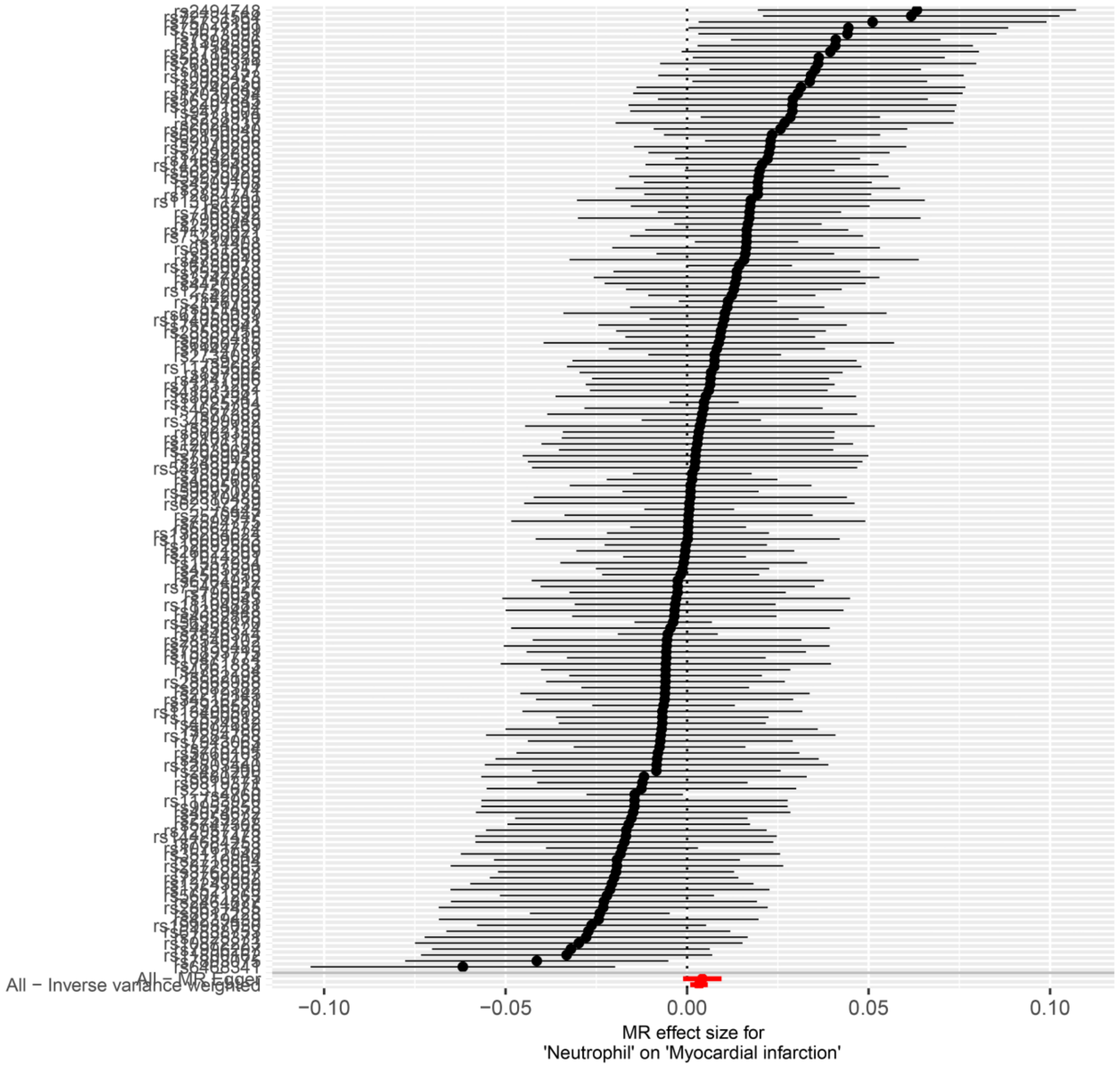

Supplementary data. Figure S1. The forest map, the bottom red line, reflects the effect of neutrophils on the risk of the development of myocardial infarction under the inverse variance weighting approach.

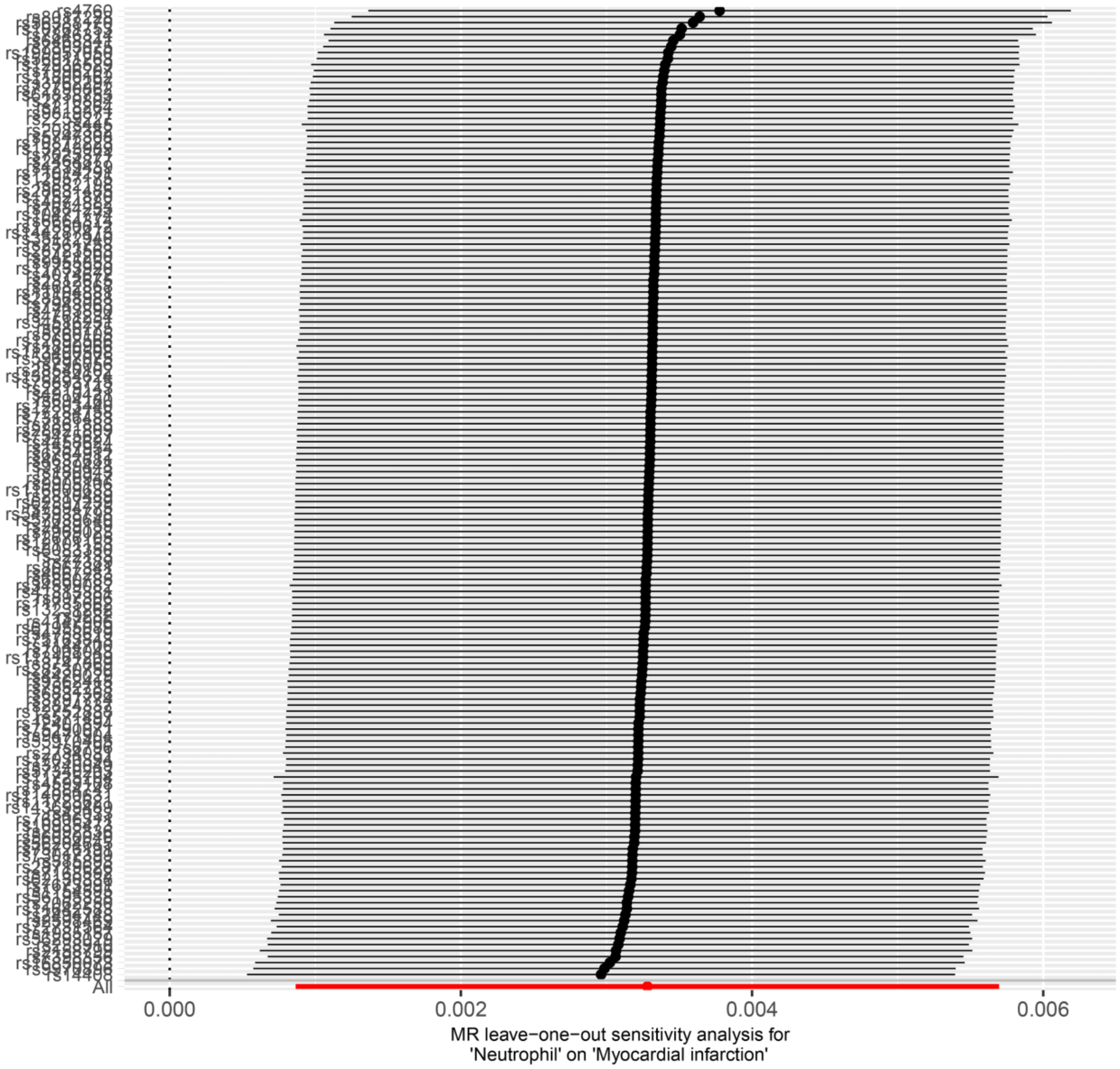

Supplementary data. Figure S2. In a leave-one-out analysis, all greater than 0 prove reliable results.

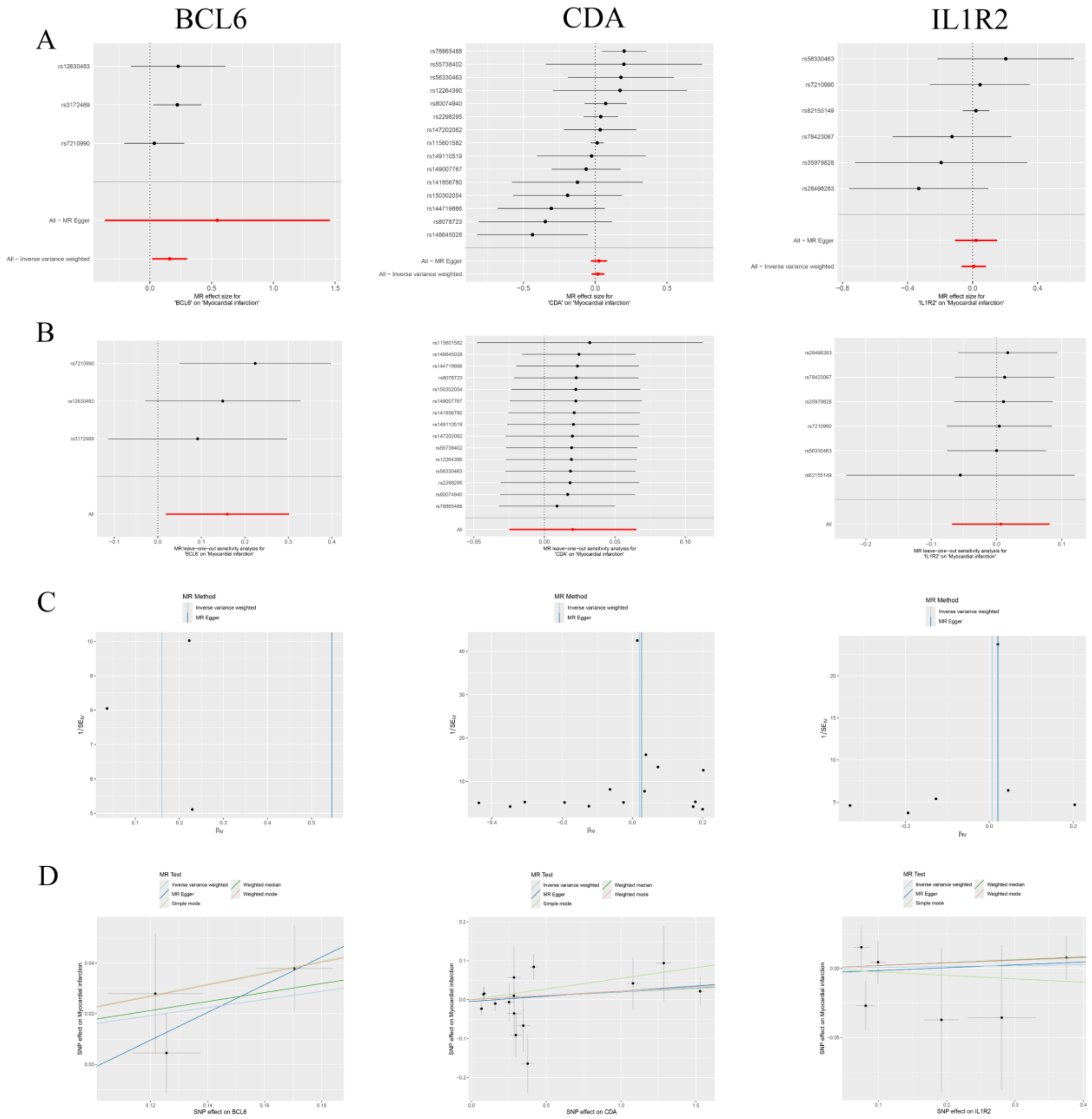

Supplementary data. Figure S3. The causal relationship between 3 hub neutrophil coexpressed genes and myocardial infarction (MI). (A) Forest plot of 3 hub neutrophil coexpressed genes associated SNPs with the risk of MI. The x-axis shows the Mendelian randomization (MR) effect size for 3 hub neutrophil coexpressed genes associated with SNPs on MI. The y-axis shows the analysis for each and the total SNPs using inverse variance weighting (IVW) methods. Dark dots: the single SNP effect (beta value). Red dot: the total SNP effect (beta value). Horizontal cross lines: standard error (SE). The vertical dotted line denotes the beta of 0. (B) MR leave-one-out sensitivity analysis for candidate genes. The x-axis represents the impact of SNPs on exposure, and the y-axis represents the impact of SNPs on outcomes. A slope greater than 0 indicates that the exposure factor is an unfavorable factor for the outcome. (C) Funnel plot of Mendelian randomization results. Funnel plots for overall heterogeneity of MR estimates for the effect of 3 hub neutrophil coexpressed genes on MI. (D) Individual estimates

about the causal impact of 3 hub neutrophil coexpressed genes on MI. The x-axis shows the single SNP (single nucleotide polymorphism) effect (beta value: the regression coefficient based on genes raising effect allele, dark dots) and standard error (SE, horizontal cross lines) of 3 hub neutrophil coexpressed genes associated SNPs, respectively. The y-axis shows the single SNP effect (beta value, dark dots) and standard error (SE, vertical cross lines) of gene-associated SNPs on MI risk. The regression lines for IVW, weighted median, MR-Egger, weighted mode, and simple mode are shown.
